# Supplementary material for: A universal reading network and its modulation by writing system and reading ability in French and Chinese children
Source: eLife. 2020 Oct 29;9:e54591. doi: 10.7554/eLife.54591 (PMC7669264; doi:10.7554/eLife.54591)
Supplement: Supplementary file 1. [file elife-54591-supp1.docx]

**S1 Table.** **Demography and performance on literacy tests for Chinese children**

|  | **Typical readers** | **Poor readers** | ***t*** | ***p*** |
| --- | --- | --- | --- | --- |
| Sample size | 24 | 24 |  |  |
| Age (months) | 123 (11) | 123 (10) | < 1 | n.s. |
| Sex | 13M/11F | 16M/8F | χ2 = 0.78 | n.s. |
| Raven non-verbal intelligence ^a^ | 72.08 (14.96) | 71.88 (15.94) | < 1 | n.s. |
| Chinese character recognition test ^b^ | 0.67 (0.09) | -1.74 (0.49) | 18.04 | **< 0.001** |
| Chinese comprehension test ^b^ | 0.46 (0.63) | -0.57 (0.60) | 5.76 | **< 0.001** |
| Character reading test ^b^ | 12.25 (2.69) | 6.61 (2.98) | 6.82 | **< 0.001** |
| Chinese phonological awareness test ^b^ | 44.05 (7.90) | 34.70 (11.38) | 3.19 | **0.003** |
| Digit cancellation test ^b^ | 0.32 (0.16) | -0.39 (0.17) | 3.04 | **0.004** |

^a^ Percentile; ^b^ Standard scores;
