# Supplementary material for: A universal reading network and its modulation by writing system and reading ability in French and Chinese children
Source: eLife. 2020 Oct 29;9:e54591. doi: 10.7554/eLife.54591 (PMC7669264; doi:10.7554/eLife.54591)
Supplement: Supplementary file 2. [file elife-54591-supp2.docx]

**S2 Table. Demography and performance on literacy tests for French children**

|  | **Typical readers** | **Poor readers** | ***t*** | ***p*** |
| --- | --- | --- | --- | --- |
| Sample size | 24 | 24 |  |  |
| Age (months) | 123 (11) | 123 (10) | < 1 | n.s. |
| Sex | 13M/11F | 16M/8F | χ2 = 0.78 | 0.376 |
| EVALEC ^b^ | 31.22 (3.69) | 23.33 (6.09) | 4.18 | **< 0.001** |
| L’alouette ^b^ | 0.73 (0.81) | -2.16 (0.52) | 14.66 | **< 0.001** |
| LUM ^b^ | 81.89 (13.17) | 25.76 (14.29) | 12.67 | **< 0.001** |

^a^ Percentile; ^a^ Standard scores;

EVALEC: Phoneme deletion test;

L’alouette: A standardized reading fluency test to detect dyslexia in French speaking children;

LUM: Number of words read in 1 min;
