# Supplementary material for: A universal reading network and its modulation by writing system and reading ability in French and Chinese children
Source: eLife. 2020 Oct 29;9:e54591. doi: 10.7554/eLife.54591 (PMC7669264; doi:10.7554/eLife.54591)
Supplement: Supplementary file 3. [file elife-54591-supp3.docx]

**S3 Table. Regions of significant activations for each visual category vs the two others in each group (individual voxel p=0.001, cluster-level FWE corrected)**

| **Region** | **MNI**  **coordinates** | **Peak**  ***p*-value** | **Peak**  **z-value** |
| --- | --- | --- | --- |
| **Chinese typical readers** |  |  |  |
| **Words > others** |  |  |  |
| Left middle temporal gyrus | -57 -39 6 | 1.89e-7 | 5.08 |
| Left precentral | -39 -3 42 | 1.58e-6 | 4.66 |
|  | -51 6 45 | 1.83e-6 | 4.63 |
| Left superior temporal pole | -51 15 -9 | 7.18e-7 | 4.82 |
| Supplementary motor area | -3 9 57 | 9.21e-7 | 4.77 |
|  | 0 18 45 | 9.96e-5 | 3.72 |
| Right precentral | 45 6 51 | 3.59e-5 | 3.97 |
| Left inferior parietal lobule | -27 -60 39 | 6.67e-5 | 3.82 |
| **Faces > others** |  |  |  |
| Left amygdala/ hippocampus | -27 -3 -18 | 1.27e-8 | 5.57 |
| Right amygdala/ hippocampus | 21 -6 -15 | 8.03e-8 | 5.24 |
| Right fusiform gyrus | 42 -51-21 | 1.7e-7 | 5.10 |
| Right inferior occipital gyrus | 30 -90 -9 | 5.58e-7 | 4.87 |
| **Houses > others** |  |  |  |
| Left fusiform gyrus | -27 -48 -9 | 9.73e-12 | 6.71 |
| Right fusiform gyrus | 24 -78 -12 | 1.01e-8 | 5.61 |
| **Chinese poor readers** |  |  |  |
| **Words > others** |  |  |  |
| Right precentral | -48 12 27 | 7.14e-9 | 5.67 |
| Left middle temporal gyrus | -51 -54 0 | 9.34e-6 | 4.28 |
| Left inferior parietal lobule | -30 -51 42 | 4.71e-6 | 4.43 |
| **Faces > others** |  |  |  |
| Right fusiform gyrus | 42 -51-21 | 1.01e-8 | 5.61 |
|  | 39 -45 -15 | 1.12e-6 | 4.73 |
| **Houses > others** |  |  |  |
| Right fusiform gyrus | 33 -30 -24 | 3.74e-9 | 5.78 |
| Left fusiform gyrus | -30 -45 -9 | 1.79e-8 | 5.51 |
| **French typical readers** |  |  |  |
| **Words > others** |  |  |  |
| Left fusiform gyrus | -54 -66 -12 | 5.04e-7 | 4.89 |
|  | -48 -45 -15 | 1.58e-6 | 4.66 |
| Supplementary motor area | 0 9 54 | 6.17e-7 | 4.85 |
| Left precentral | -42 3 30 | 2.01e-6 | 4.61 |
| Left inferior parietal lobule | -54 -33 51 | 2.91e-5 | 4.02 |
| **Faces > others** |  |  |  |
| Right fusiform gyrus | 45 -51 -21 | 2.61e-9 | 5.84 |
|  | 39 -66 -12 | 2.66e-8 | 5.44 |
| Left amygdala/ hippocampus | 21 -9 -15 | 3.18e-7 | 4.98 |
| Left fusiform gyrus | -36 -69 -15 | 3.56e-6 | 4.49 |
| Right amygdala/ hippocampus | -24 -3 -21 | 4.71e-6 | 4.43 |
| **Houses > others** |  |  |  |
| Left fusiform gyrus | -27 -78 -12 | 7.92e-12 | 6.74 |
| Right fusiform gyrus | 27 -48 -6 | 3.32e-9 | 5.80 |
| Left superior occipital gyrus | -18 -96 15 | 9.55e-9 | 5.62 |
| **French poor readers** |  |  |  |
| **Words > others** |  |  |  |
| Left precentral | -42 0 27 | 2.67e-5 | 4.04 |
| **Faces > others** |  |  |  |
| Right fusiform gyrus | 42 -48 -24 | 2.13e-8 | 5.48 |
| Left fusiform gyrus | -39 -48-21 | 6.46e-8 | 5.28 |
| Left fusiform gyrus | -36 -87 -12 | 1.11e-7 | 5.18 |
| Left calcarine | -3 -87 -9 | 1.23e-7 | 5.16 |
| Right fusiform gyrus | 45 -60 15 | 1.24e-6 | 4.71 |
| Right precuneus | 6 -45 60 | 2.79e-5 | 4.03 |
| **Houses > others** |  |  |  |
| Left fusiform gyrus | -27 -48 -9 | 7.57e-9 | 5.66 |
| Right fusiform gyrus | 30 -48 -12 | 8.02e-9 | 5.65 |
| Right precuneus | 24 -57 27 | 1.12e-5 | 4.24 |
