# Supplementary material for: A universal reading network and its modulation by writing system and reading ability in French and Chinese children
Source: eLife. 2020 Oct 29;9:e54591. doi: 10.7554/eLife.54591 (PMC7669264; doi:10.7554/eLife.54591)
Supplement: Supplementary file 4. [file elife-54591-supp4.docx]

**S4 Table. Summary of activation foci in meta-analyses of dyslexia in alphabetic languages**

| Regions | Study | Coordinates  (MNI) | | |
| --- | --- | --- | --- | --- |
|  |  | x | y | z |
| Left Fusiform Gyrus | Richlan et al., 2009 | -46 | -50 | -16 |
|  | Maisog et al.,2008 | -48 | -42 | -22 |
|  | Richlan et al., 2011 | -40 | -42 | -20 |
|  | Linkersdorfer et al., 2012 | -44 | -50 | -16 |
| Inferior Parietal Lobule | Richlan et al., 2009 | -52 | -46 | 44 |
|  | Richlan et al., 2011 | -40 | -48 | 42 |
| Inferior Frontal Gyrus opercularis | Richlan et al., 2009 | -46 | 16 | 6 |
|  | Linkersdorfer et al., 2012 | -54 | 14 | 12 |
|  |  | -56 | 20 | 6 |
|  |  | -42 | 8 | 14 |
|  |  | -46 | 18 | 6 |
| Inferior Temporal Gyrus | Richlan et al., 2009 | -52 | -62 | -8 |
|  | Maisog et al., 2008 | -48 | -58 | -10 |
| Posterior Superior Temporal Gyrus | Richlan et al., 2009 | -52 | -44 | 20 |
|  | Linkersdorfer et al., 2012 | -52 | -42 | 22 |
| Superior Temporal Gyrus | Richlan et al., 2009 | -52 | -28 | 0 |
|  | Maisog et al., 2008 | -52 | -36 | 8 |
|  | Linkersdorfer et al., 2012 | -52 | -32 | 4 |
|  |  | -44 | -26 | 8 |
| Middle Temporal Gyrus | Richlan et al., 2009 | -60 | -56 | 2 |
|  | Richlan et al., 2011 | -60 | -54 | 2 |
|  | Linkersdorfer et al., 2012 | -52 | -50 | 12 |
|  |  | -46 | -46 | 4 |
| Supramarginal Gyrus | Richlan et al., 2011 | -52 | -48 | 32 |
|  | Linkersdorfer et al., 2012 | -44 | -44 | 40 |
|  |  | -58 | -48 | 38 |
|  |  | -32 | -40 | 34 |
|  |  | -60 | -42 | 30 |
| Precentral Gyrus | Linkersdorfer et al., 2012 | -42 | 4 | 36 |
| Superior Parietal Lobule | Linkersdorfer et al., 2012 | -34 | -62 | 50 |
