# Supplementary material for: A universal reading network and its modulation by writing system and reading ability in French and Chinese children
Source: eLife. 2020 Oct 29;9:e54591. doi: 10.7554/eLife.54591 (PMC7669264; doi:10.7554/eLife.54591)
Supplement: Supplementary file 5. [file elife-54591-supp5.docx]

**S5 Table. Summary of foci in meta-analyses of reading in Chinese**

| Regions | Study | Coordinates  (MNI) | | |
| --- | --- | --- | --- | --- |
|  |  | x | y | z |
| Left Middle Frontal Gyrus | Tan et al., 2005 | -46 | 18 | 28 |
|  | Wu et al., 2012 | -48 | 14 | 32 |
|  |  | -46 | 18 | 28 |
|  | Zhu et al., 2012 | -40 | 18 | 26 |
|  |  | -44 | 16 | 32 |
| Right Inferior Occipital Gyrus | Bolger et al., 2005 | 29 | -89 | -9 |
|  | Tan et al., 2005 | 36 | -82 | -12 |
|  | Wu et al., 2012 | 28 | -86 | 0 |
|  |  | 26 | -92 | -12 |
|  | Zhu et al., 2012 | 26 | -86 | 0 |
| Right Fusiform | Bolger et al., 2005 | 33 | -67 | -14 |
|  | Tan et al., 2005 | 34 | -60 | -18 |
|  | Wu et al., 2012 | 44 | -58 | -12 |
