# Supplementary material for: A universal reading network and its modulation by writing system and reading ability in French and Chinese children
Source: eLife. 2020 Oct 29;9:e54591. doi: 10.7554/eLife.54591 (PMC7669264; doi:10.7554/eLife.54591)
Supplement: Supplementary file 6. [file elife-54591-supp6.docx]

**S6 Table.** Distance between individual center of 10 most activated voxels and group peaks.

|  | **Chinese** | |  | **French** | |
| --- | --- | --- | --- | --- | --- |
|  | **Typical readers** | **Poor readers** |  | **Typical readers** | **Poor readers** |
| **Reading areas** | | | | | |
| FFG | 8.07 (1.85) | 7.93 (2.44) |  | 8.97 (1.53) | 8.91 (1.48) |
| MFG | 7.16 (1.95) | 7.01 (2.03) |  | 7.85 (1.85) | 7.56 (1.90) |
| STS | 7.34 (2.32) | 8.11 (1.61) |  | 8.81 (1.67) | 8.36 (2.44) |
| PCG | 6.74 (2.14) | 7.58 (1.69) |  | 6.70 (2.37) | 6.85 (2.74) |
| pSTG | 7.03 (2.03) | 6.94 (2.05) |  | 7.43 (1.69) | 7.95 (1.91) |
| SPL | 8.59 (2.07) | 8.18 (2.85) |  | 8.51 (1.53) | 8.56 (2.06) |
| **Face areas** | | | | | |
| L.FFA | 8.65 (1.49) | 8.29 (1.97) |  | 8.18 (1.92) | 7.87 (1.59) |
| R.FFA | 7.71 (1.93) | 7.57 (1.88) |  | 7.94 (1.75) | 8.32 (1.61) |

Note: Neither the main effect of reading ability nor the interaction with language was significant.
