# Supplementary material for: A universal reading network and its modulation by writing system and reading ability in French and Chinese children
Source: eLife. 2020 Oct 29;9:e54591. doi: 10.7554/eLife.54591 (PMC7669264; doi:10.7554/eLife.54591)
Supplement: Transparent reporting form [file elife-54591-transrepform.pdf]

eLIFE

1st Floor
24 Hills Road
Cambridge CB2 1JP, UK

P 01223 855340
W elifesciences.org
T @elife

eLife's transparent reporting form

We encourage authors to provide detailed information *within their submission* to facilitate the interpretation and replication of experiments. Authors can upload supporting documentation to indicate the use of appropriate reporting guidelines for health-related research (see EQUATOR Network), life science research (see the BioSharing Information Resource), or the ARRIVE guidelines for reporting work involving animal research. Where applicable, authors should refer to any relevant reporting standards documents in this form.

If you have any questions, please consult our Journal Policies and/or contact us: editorial@elifesciences.org.

Sample-size estimation

- You should state whether an appropriate sample size was computed when the study was being designed
- You should state the statistical method of sample size computation and any required assumptions
- If no explicit power analysis was used, you should describe how you decided what sample (replicate) size (number) to use

Please outline where this information can be found within the submission (e.g., sections or figure legends), or explain why this information doesn't apply to your submission:

Our sample size (96 children in total with 24 in each group) was chosen according to the previous literature. fMRI studies on children with developmental disorders typically include close to 25 participants, which can be seen as a general minimum.

We have to admit that our main conclusions in the current study are based on a null effect (i.e. no difference between Chinese and French dyslexics).
However, there are still many positive, significant and interesting discoveries in our study. Besides, we are in the best possible experimental situation to perform a cross-linguistic comparison: our groups are perfectly paired, very homogeneous in age and comprise a large number of children (24 in each group, for a total of 96 children; this is no small feat when considering the constraints that face such developmental studies). We used the same experimental task and brain imaging acquisition parameters in France and China, thus controlling for many confounding factors. Furthermore, our study is obviously sufficiently powered, since we have positive effects of reading performance and language. Furthermore, we present numerous analyses aimed at overcoming a possible lack of power at the whole brain level, particularly by focusing on regions of interest, and by performing peak location and multivariate analyses. Finally, we also did Bayesian analyses on the same data. All these analyses confirm our conclusion that neural bases of both reading acquisition and reading impairment in two very different languages (French and Chinese) are largely universal.

Replicates

- You should report how often each experiment was performed
- You should include a definition of biological versus technical replication

- The data obtained should be provided and sufficient information should be provided to indicate the number of independent biological and/or technical replicates
- If you encountered any outliers, you should describe how these were handled
- Criteria for exclusion/inclusion of data should be clearly stated
- High-throughput sequence data should be uploaded before submission, with a private link for reviewers provided (these are available from both GEO and ArrayExpress)

Please outline where this information can be found within the submission (e.g., sections or figure legends), or explain why this information doesn't apply to your submission:

fMRI experiments are typically only performed once on a specific task, hence this information does not apply to this submission.

Statistical reporting

- Statistical analysis methods should be described and justified
- Raw data should be presented in figures whenever informative to do so (typically when N per group is less than 10)
- For each experiment, you should identify the statistical tests used, exact values of N, definitions of center, methods of multiple test correction, and dispersion and precision measures (e.g., mean, median, SD, SEM, confidence intervals; and, for the major substantive results, a measure of effect size (e.g., Pearson's r, Cohen's d)
- Report exact p-values wherever possible alongside the summary statistics and 95% confidence intervals. These should be reported for all key questions and not only when the p-value is less than 0.05.

Please outline where this information can be found within the submission (e.g., sections or figure legends), or explain why this information doesn't apply to your submission:

Statistical analysis methods are described throughout the Result section, and more details can be found in the Materials & Methods section.

Because some of our analyses evaluated the null hypothesis of no difference across the two languages, we also performed Bayesian analyses on the same data and reported the Bayes Factor in the Result Section.

(For large datasets, or papers with a very large number of statistical tests, you may upload a single table file with tests, Ns, etc., with reference to sections in the manuscript.)

Group allocation

- Indicate how samples were allocated into experimental groups (in the case of clinical studies, please specify allocation to treatment method); if randomization was used, please also state if restricted randomization was applied
- Indicate if masking was used during group allocation, data collection and/or data analysis

Please outline where this information can be found within the submission (e.g., sections or figure legends), or explain why this information doesn't apply to your submission:

Group allocation and methods for identifying Chinese and French dyslexia are reported in the Participants Section.

Additional data files ("source data")

- We encourage you to upload relevant additional data files, such as numerical data that are represented as a graph in a figure, or as a summary table
- Where provided, these should be in the most useful format, and they can be uploaded as "Source data" files linked to a main figure or table
- Include model definition files including the full list of parameters used
- Include code used for data analysis (e.g., R, MatLab)
- Avoid stating that data files are "available upon request"

Please indicate the figures or tables for which source data files have been provided:

eLife Sciences Publications, Ltd is a limited liability non-profit non-stock corporation incorporated in the State of Delaware, USA, with company number 5030732, and is registered in the UK with company number FC030576 and branch number BR015634 at the address 1st Floor, 24 Hills Road, Cambridge CB2 1JP | August 2014

(1) Summary behavior data of our participants can be found in table 1, Supplementary file 1 and 2.
(2) Summary tables of imaging results can be found in Table 2,3 and Supplementary file 3.
(3) Source data files have been provided for Fig 1, Fig2, Fig 3, Fig4, Fig 6 in the manuscript and supplementary files.
(4) The processed data to generate figures in this manuscript will be shared in the Open Science Framework (the procedure is on-going).
